# Supplementary material for: Biogenic non-crystalline U(IV) revealed as major component in uranium ore deposits
Source: Nat Commun. 2017 Jun 1;8:15538. doi: 10.1038/ncomms15538 (PMC5461479; doi:10.1038/ncomms15538)
Supplement: Supplementary Information — Supplementary figures, supplementary tables and supplementary references. [file ncomms15538-s1.pdf]

Supplementary Figure 1.

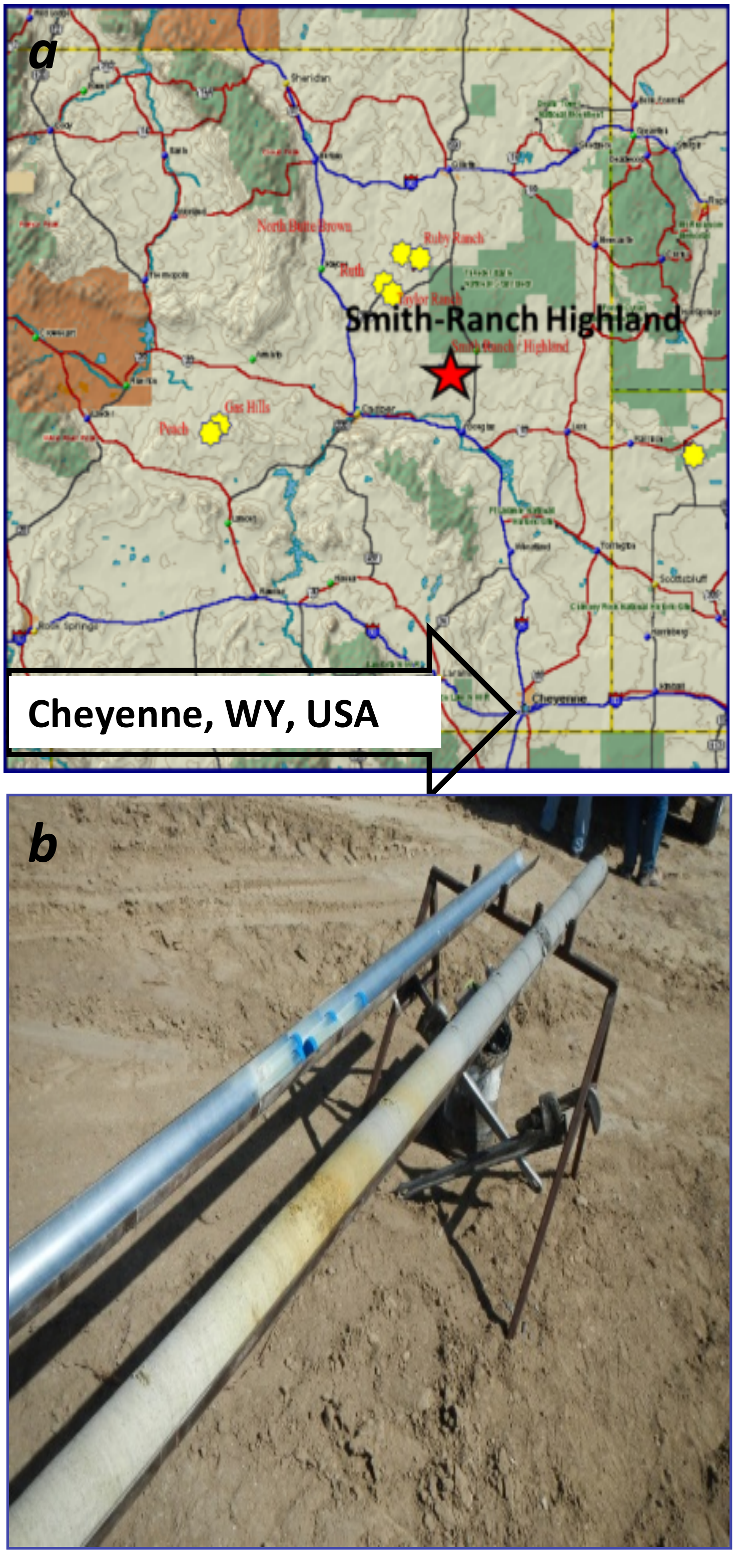

Supplementary Figure 1. a) Area map *(pic courtesy: James Clay, Cameco Resources)*, b) core drilled from 200 m-bgs.

Supplementary Figure 2.

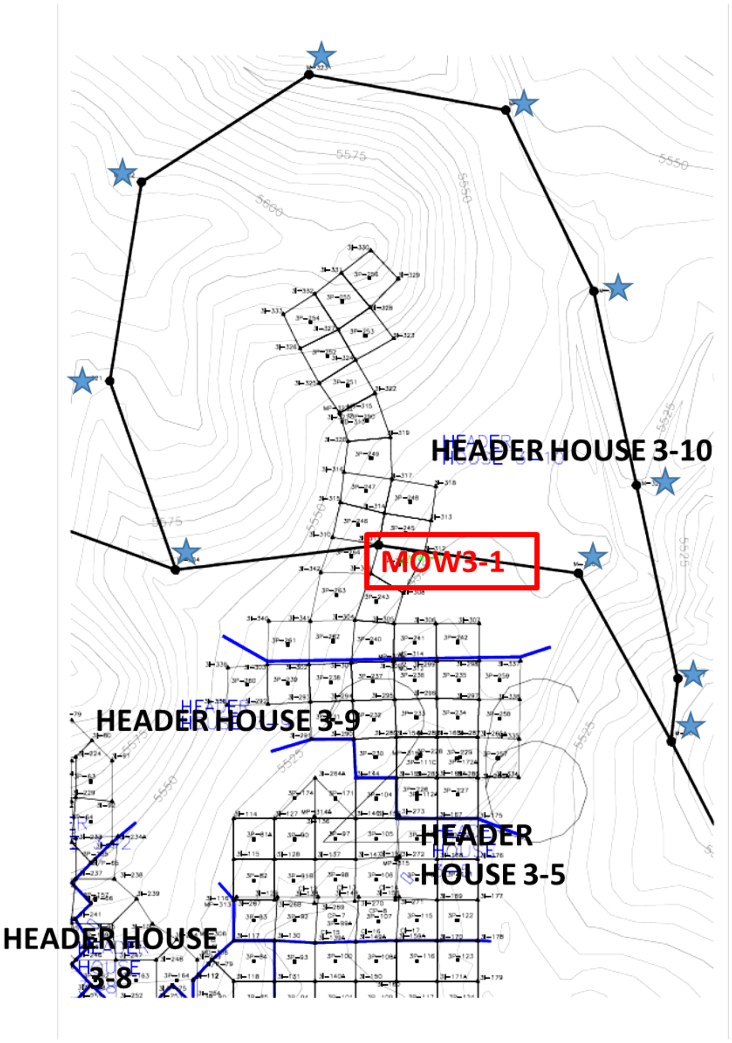

Supplementary Figure 2. Location of Mine Unit 3 extension along with the header houses and monitoring wells in the vicinity. The location of the red box MOW3-1 indicates the location from where the core was drilled. Blue stars indicate the locations of the monitoring wells. The header house locations are also indicated in the figure (*not to scale*). Base map used with the permission of Cameco Corporation.

Supplementary Figure 3.

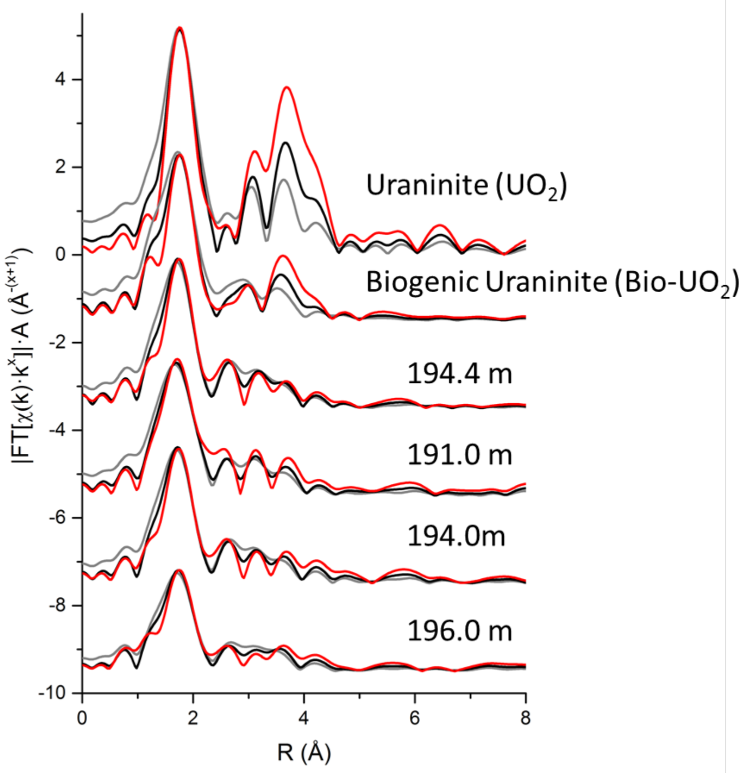

Supplementary Figure 3. Magnitude of Fourier Transform using *k*-weight of 1 (grey), 2 (black), and 3 (red) are shown (top to bottom) for crystalline uraninite (UO_2), biogenic uraninite, 194.4 m, 191.0 m, 194.0 m, and 196.0 m sediment samples in groups with each sample offset below for clarity. The *k*-weight 1 and 2 spectra have been multiplied by a constant A so that the first shell signal between 1 and 2 Å has the same amplitude. In this way the nature of the second and additional shell signals can be compared. Second shell signals from atoms with similar number of electrons to the first shell O (such as O and C) will also scale similarly so that the amplitudes are similar for all three spectra, as is the case for the sediment spectra. The second shell signal form uraninite is from U with many more electrons. This U signal will have more amplitude in Fourier transforms with higher *k*-values so that the amplitude of this signal is emphasized with *k*-weight of 2 and 3 compared to *k*-weight of 1. As seen in the graph the U--U signal between 3 to 5 Å grows dramatically.

Supplementary Figure 4.

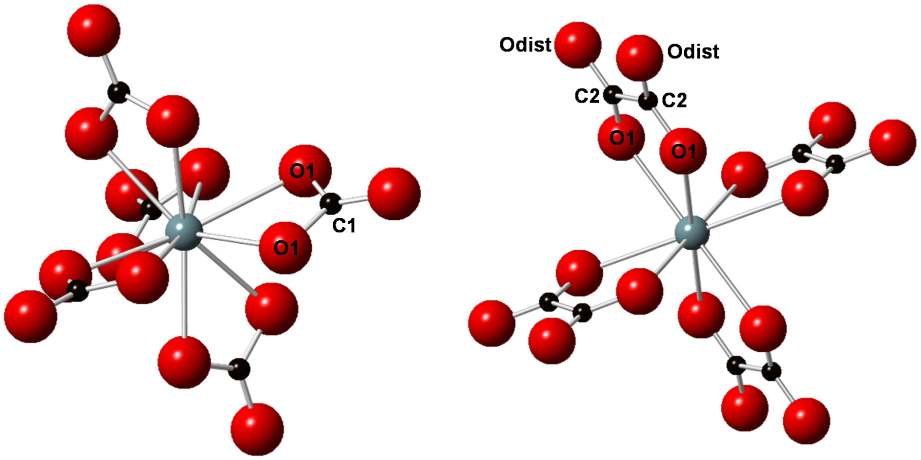

Supplementary Figure 4. Ball-and-stick model of the two structures used to build the model for the EXAFS spectra. The model includes U-O1 found in both configurations, U--C1 indicative of a carbonate environment and U--C2 and U--O_{dist} indicative of an oxalate environment. Only one ligand is labeled in each structure for clarity.

Supplementary Figure 5.

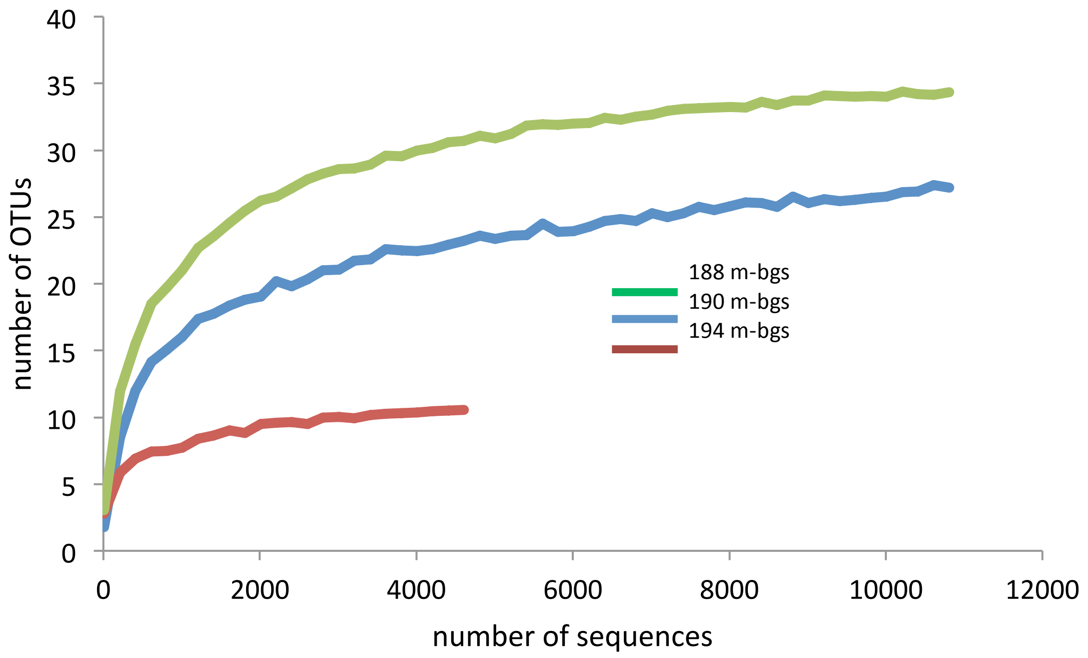

Supplementary Figure 5. Rarefaction curves show acceptable sampling depths for DNA samples analyzed for the 3 roll-front samples.

Supplementary Table 1. Sample identification and summary of analysis performed on each depth.

**Sample depths (m-bgs)**	**TIC/TOC**	**U Sequential extractions**	**XRD**	**U-EXAFS**	**U-Isotope**	**16S rRNA**
187.8			X			*
188.0						X
188.3			X			*
190.0						X
191.0	X	X	X	X	X	*
191.4			X			*
193.5			X			*
193.8	X		X	X		*
194.0	X	X		X	X	X
194.4				X		*
195.0			X			*
196.0	X	X	X	X	X	*
198.0		X	X		X	*
199.0			X			*

The asterisk (*) symbol represents that there was not enough biomass to perform 16S rRNA analysis on these sediments.

Supplementary Table 2. U concentrations (mg kg^{-1}) obtained from ICPMS analysis of sequential extractions and digests of sediment samples at given depths.

Depth (m-bgs)	Total U	Exchangeable U	Carbonate bound U	U in Fe/Mn oxide	Organically bound U	Residual (clay) U
191	231.86	2.96	59.38	7.66	69.78	92.08
194	164.11	1.52	63.59	6.482	41.80	50.72
196	237.96	2.11	65.87	12.41	64.05	93.52
198	190.00	2.28	64.94	6.06	67.69	49.03

Supplementary Table 3. X-ray diffraction (XRD) data for sediments at various depths (m-bgs) on the <2mm size fraction. Any phases less than approximately 1% by weight were not detectable by XRD.

**Sample depth (m-bgs)**	**187.8**	**188.3**	**190**	**191**	**191.4**	**193.5**	**193.8**	**195**	**196**	**199**
**Mineral**	Wt (%)	Wt (%)	Wt (%)	Wt (%)	Wt (%)	Wt (%)	Wt (%)	Wt (%)	Wt (%)	Wt (%)
**NON-CLAYS**										
**Quartz**	51.5	41.4	68.3	69.2	47.8	50.6	57.8	50.3	72.8	49.7
**Kspar (ordered Microcline)**	24.9	34.4	14.7	11.9	18.3	16.2	13.8	25.9	9.1	29.7
**Plagioclase (albite, var. cleavelandite)**	15.1	16.1	7.7	5.9	10.5	6.9	6.0	13.8	4.7	14.4
**Calcite**	0.0	0.0	0.0	0.0	13.5	0.0	0.1	0.0	0.0	0.0
**Pyrite**	0.0	0.0	0.0	0.1	0.2	0.2	0.1	0.1	0.1	0.1
**Total non-clays**	91.5	91.9	90.7	87.1	90.3	73.9	77.9	90.0	86.7	93.9
**CLAYS**										
**Kaolinite (disordered)**	1.5	1.7	2.6	3.7	2.4	2.2	2.4	1.9	3.3	2.0
**Smectite (Ca-Kinney montmorillonite)**	0.1	0.0	1.7	1.5	1.1	10.9	6.9	1.5	2.3	0.0
**Illite (1Md)**	5.7	4.4	3.5	3.9	4.2	8.4	7.9	6.4	6.3	2.2
**Muscovite (2M1)**	1.1	1.9	1.5	3.7	2.0	4.6	4.9	0.2	1.3	1.9
**Total clays**	8.5	8.1	9.3	12.9	9.7	26.1	22.1	10.0	13.3	6.1
**TOTAL**	100.0	100.0	100.0	100.0	100.0	100.0	100.0	100.0	100.0	100.0

Supplementary Table 4. Percentages of total inorganic (TIC) and organic carbon (TOC) for selected sediment depths

Sediment depth (m-bgs)	% Total Inorganic Carbon (TIC)	% Total Organic Carbon (TOC)
191.0	1.91	4.56
193.8	6.32	3.67
194.0	1.78	4.65
196.0	1.89	4.92

Supplementary Table 5. $^{38}U/^{235}U$ ratios and corresponding δ ^{238}U values (in per mil) for sediment samples presented in Fig. 3. δ $^{238}U_{CRM-112A}$ = 137.837‰ (Richter et al., 2010)[1].

Sediment Depth (m-bgs)	Total U (mg kg^{-1})	$^{238}U/^{235}U$	$\delta^{238}U$(‰)	2 SD
191	231.86	137.83	0.084	0.041
194	164.11	137.84	0.1581	0.10
196	237.96	137.73	-0.68	0.09
198	190.00	137.81	-0.057	0.025

Supplementary Table 6. Summary of taxonomy at the genus level from 16S rRNA analysis, in percent for selected sediment depths (m-bgs). "Other" indicates the organism was less than 97% similar to a classified organism at the genus level.

Genus	188.0 m-bgs	190.0 m-bgs	194.0 m-bgs
Betaproteobacteria, other	25.3	0.5	0.0
Geothrix	18.6	0.0	0.0
Thiobacillus	12.6	0.2	0.0
Acinobacteria, other	5.2	0.2	0.0
Patulibacter	3.9	0.2	0.0
Pseudomonas	3.1	11.2	31.3
Acinetobacter	2.3	52.8	0.1
Leifsonia	1.3	0.0	0.0
Solirubrobacter	1.2	0.0	0.0
Acinetobacter	1.0	21.1	0.0
Gemmatimonas	0.8	0.6	0.0
Bdellovibrio	0.7	0.0	0.0
Streptosporangium	0.6	0.0	0.0
Paracraurococcus	0.5	0.0	0.0
Clostridium	0.5	0.0	0.0
Rhodocyclaceae other	0.5	0.0	0.0
Propionibacterium	0.4	1.2	6.4
Streptococcus	0.4	0.6	0.9
Bellilinea	0.3	0.3	0.0
Sphingobacteriales other	0.3	0.0	0.0
Bradyrhizobium	0.3	0.0	0.0
Acidobacterium	0.3	0.7	0.0
Bradyrhizobium	0.3	0.0	0.0
Aerococcus	0.3	0.0	0.0
Pseudomonas	0.2	0.4	0.0
Halomonas	0.2	0.0	0.0
Opitutus	0.2	0.0	0.0
Sphingomonas	0.2	0.5	0.0
Corynebacterium	0.2	0.3	0.0
Burkholderiales other1	0.2	0.9	0.0
Rhizobiales other	0.1	0.0	0.0
Thiorhodospira	0.1	0.0	0.0
Desemzia	0.1	0.0	0.0
Geobacter	0.1	0.3	0.0
Staphylococcus	0.1	0.2	0.9
Parvularcula	0.1	0.0	0.0
Herbaspirillum	0.1	0.5	0.0
Jeotgalicoccus	0.1	0.0	0.0

Moraxella	0.1	0.0	0.0
Jeotgalicoccus	0.1	0.0	0.0
Cohnella	0.1	0.0	0.0
Methylococcus	0.1	0.0	0.0
Kocuria	0.1	0.0	0.0
Nitrospira	0.1	0.0	0.0
Turicibacter	0.1	0.0	0.0
Unknown	0.1	0.0	0.0
Acetivibrio	0.1	0.0	0.0
Burkholderiales other2	0.0	0.3	0.0
Actinomycetales other	0.0	0.0	27.3
Clostridium	0.0	0.0	0.0
Thermodesulfovibrio	0.0	0.1	0.0
Microbacteriaceae other	0.0	0.0	0.6
Arthrobacter	0.0	0.5	0.4
Arthrobacter	0.0	0.0	0.1
Micrococcus	0.0	0.0	1.0
Mycobacterium	0.0	0.0	0.7
Nocardioides	0.0	0.0	0.2
Thermomonosporaceae, other	0.0	0.0	3.3
Porphyromonas	0.0	0.2	0.0
Prevotella	0.0	0.2	0.0
Bacillus	0.0	0.0	1.2
Paenibacillus	0.0	0.2	2.5
Lactobacillus	0.0	0.0	0.1
Blautia	0.0	0.0	0.5
Ruminococcus	0.0	0.0	1.3
Clostridiales other	0.0	0.1	0.0
Megamonas	0.0	0.1	0.0
Fusobacterium	0.0	0.8	0.0
Roseobacter	0.0	0.3	0.0
Sphingobium	0.0	0.9	15.5
Sphingomonas	0.0	0.2	0.0
Acidovorax	0.0	0.2	1.7
Malikia	0.0	0.0	0.5
Neisseria	0.0	0.1	0.0
Nitrosospira	0.0	0.1	0.0
Rickettsiella	0.0	0.2	0.0
Coxiellaceae other	0.0	0.2	0.0
**Sum of all taxa not identified to order level**	**16.5**	**2.8**	**3.6**

Supplementary Table 7. Description of the EXAFS model for sample depths (m-bgs) 191.0, 193.8, 194.0 and 194.4 m-bgs and best fit values for EXAFS parameters R and σ^2.

**Path**	**CN**	**R (Å)**	**σ^2 ($\cdot 10^{-3}$ Å^2)**	**ΔE (eV)**	**Description**
U-O_{ax}	N-O_{ax}	1.76 ± 0.01	1.0*	3 ± 0.8	Axial oxygen atoms from uranyl
U-O1	N-O1	2.37 ± 0.01	20 ± 2	3 ± 0.8	Oxygen atoms bound to U(VI) and U(IV)
U-C1	N-C1	2.81 ± 0.01	1 ± 7	3 ± 0.8	Carbon atom from bidentate carbon group
U-C2	N-C2	3.43 ± 0.03	6 ± 6	3 ± 0.8	Carbon atoms from oxalate group
U-Oeq-C2	N-C2	3.64 ± 0.03	6 ± 6	3 ± 0.8	Multiple scattering from O1 and C2
U-O_{dist}	N-C2	4.38 ± 0.07	7 ± 6	3 ± 0.8	Distant oxygen atom that is part of oxalate-like group
U-C2-O_{dist}	N-C2*2	4.39 ± 0.07	7 ± 6	3 ± 0.8	Multiple scattering from C2 and O_{dist}
U-C2-O_{dist}-C2	N-C2	4.41 ± 0.07	7 ± 6	3 ± 0.8	Multiple scattering from C2 and O_{dist}
U-U	*N-U1*	*4.01 ± 0.06*	*7 ± 12*	*3 ± 0.8*	*Uranium neighbor in uraninite*

* *σ^2 for U-O_{ax} held at 0.001 Å^2*

Supplementary Table 8. Description of the EXAFS model for sample depth 196.0 m-bgs and best fit values for EXAFS parameters R and σ^2.

**Path**	**CN**	**R (Å)**	σ^2 **($\cdot 10^{-3}$ Å^2)**	**ΔE (eV)**	**Description**
U-O_{ax}	N-O_{ax}	1.76 ± 0.02	1.0*	3 ± 0.9	Axial oxygen atoms from uranyl $U^{(VI)}$
U-O1	N-O1	2.36 ± 0.01	16 ± 1	3 ± 0.9	Oxygen atoms bound to $U^{(VI)}$ and $U^{(IV)}$
U-O2	N-O2 = 8 x FU4[a] – N-O1	2.85 ± 0.01	16 ± 1	3 ± 0.9	Oxygen atoms bound to $U^{(IV)}$
U-C2	N-C2	3.39 ± 0.06	26 ± 8	3 ± 0.9	Carbon atoms from oxalate group
U-O_{eq}-C2	N-C2 x 2	3.61 ± 0.06	26 ± 8	3 ± 0.9	Multiple scattering from O1 and C2
U-O_{dist}	N-C2	4.46 ± 0.03	18 ± 6	3 ± 0.9	Distant oxygen atom that is part of oxalate group
U-C2-O_{dist}	N-C2 x 2	4.48 ± 0.03	18 ± 6	3 ± 0.9	Multiple scattering from C2 and O_{dist}
U-C2-O_{dist}-C2	N-C2	4.49 ± 0.03	18 ± 6	3 ± 0.9	Multiple scattering from C2 and O_{dist}

a: fraction of $U^{(IV)}$ as determined by 1- N-O_{ax}/2.

** σ^2 for U-O_{ax} held at 0.001 Å^2*

Supplementary Reference

1 Richter, S. *et al.* New average values for the n (238 U)/n (235 U) isotope ratios of natural uranium standards. *International Journal of Mass Spectrometry* **295**, 94-97 (2010).
