# Supplementary material for: Biogenic non-crystalline U(IV) revealed as major component in uranium ore deposits
Source: Nat Commun. 2017 Jun 1;8:15538. doi: 10.1038/ncomms15538 (PMC5461479; doi:10.1038/ncomms15538)
Supplement: Supplementary Data 1 — Ore grade printout and gamma radiation emissions of MOW3-1 [file ncomms15538-s2.docx]

1. ORE GRADE PRINTOUT OF MOW3-1

CENTURY GEOPHYSICAL CORPORATION

* * * ORE-GRADE ANALYSIS * * *

COMPANY : CAMECO RESOURCES WELL : 3674-26-MOW3-1

FIELD : 3 DATE : 05/24/13 K-FACTOR in 0.00001 : 0.5000 DEAD TIME: 3.000 USEC

CUTOFFS SELECTED:

CO1= 0.020 %

CO2= 0.030 %

CO3= 0.050 %

INTERVAL SELECTED: 1.0 FT ** ZONE 1 : 385.50 - 390.00 : ALL VALUES BELOW THE LOWEST CUTOFF VALUE.

** ZONE 2 : 542.50 - 549.00 : ALL VALUES BELOW THE LOWEST CUTOFF VALUE.

* ZONE 3 *

DEPTH TRUE DEPTH GRADE CO1 CO2 CO3 INT

[FT][FT] [%]

626.00 625.9 0.012

626.50 626.4 0.020

627.00 626.9 0.028 *

627.50 627.4 0.036 * *

628.00 627.9 0.038 * *

628.50 628.4 0.043 * *

629.00 628.9 0.063 * * *

629.50 629.4 0.090 * * *

630.00 629.9 0.125 * * *

630.50 630.4 0.149 * * *

631.00 630.9 0.103 * * *

631.50 631.4 0.102 * * *

632.00 631.9 0.094 * * *

632.50 632.4 0.119 * * *

633.00 632.9 0.202 * * * *

633.50 633.4 0.153 * * * *

634.00 633.9 0.073 * * * *

634.50 634.4 0.055 * * * 6.0 F/ 0.111%

635.00 634.9 0.036 * *

635.50 635.4 0.041 * *

636.00 635.9 0.086 * * *

636.50 636.4 0.131 * * *

637.00 636.9 0.089 * * *

637.50 637.4 0.073 * * *

638.00 637.9 0.086 * * *

638.50 638.4 0.089 * * *

639.00 638.9 0.083 * * *

639.50 639.4 0.063 * * *

640.00 639.9 0.059 * * *

640.50 640.4 0.071 * * *

641.00 640.9 0.065 * * 14.0 F/ 0.086% * 5.5 F/ 0.081%

641.50 641.4 0.024 * 15.0 F/ 0.082%

642.00 641.9 0.020

BEST 1.0 FOOT INTERVAL 0.178 AVERAGE GRADE, 0.178 GTP

- - - * * *

THRESHOLD : 0.000

WATER DEPTH (FT): 0.000

WATER FACTOR : 1.139

CASING DEPTH (FT): 0.000

CASING FACTOR : 0.000

WASTE INTERVAL (FT): 0.000





(2) Gamma Radiation Emissions (~620 to 675 feet-bgs) of MOW3-1
